# Associations between employment status, type of occupation, and mental health problems in a treatment seeking sample

**DOI:** 10.3389/fpsyg.2025.1536914

**Published:** 2025-04-30

**Authors:** Jakob Lundqvist, Martin S. Lindberg, Martin Brattmyr, Audun Havnen, Odin Hjemdal, Stian Solem

**Affiliations:** ^1^Department of Psychology, Norwegian University of Science and Technology, Trondheim, Norway; ^2^Trondheim Municipality, Health and Welfare, Trondheim, Norway; ^3^St. Olavs University Hospital, Nidaros Community Mental Health Centre, Trondheim, Norway

**Keywords:** depression, anxiety, health, occupation, employment, disability

## Abstract

**Background:**

Previous studies have not investigated psychological profiles across different types of occupations and unemployment in samples seeking mental health treatment.

**Aims:**

The main aim of the study was to explore associations between employment status, type of occupation, and mental health problems in a clinical sample.

**Methods:**

The sample consisted of 2014 participants seeking treatment at a community mental health service. Employment status and type of occupation were compared with the general Norwegian population. Logistic regression analyses (adjusting for age, sex, household income, and relationship status) explored associations between mental health (use of psychotropics, alcohol, depression, anxiety, work- and social functioning, and general health), employment status, and type of occupation.

**Results:**

Unemployed participants, individuals on work assessment allowance/other benefits, and clerical workers were overrepresented in the sample. People receiving disability benefits or work assessment allowance showed higher use of psychotropic medication, reported more anxiety and depression, and lower functioning and health. There were some significant differences between specific occupations, but these effects were relatively small.

**Conclusion:**

The findings suggested that type of occupation was less relevant to mental health outcomes, whereas different types of unemployment was clearly associated with worse mental health. Future research should address treatments integrating mental health focus along with work focus.

## Introduction

Mental illness is one of the leading causes of sickness absence and represents a major challenge for society. Norway has extraordinarily high rates of sickness absence and use of disability benefits compared with other countries, and ill-mental health is the biggest issue (Hemmings and Prinz, 2020). In Norway, people can receive sickness benefits for up to 52 weeks. Thereafter, they can apply for work assessment allowance if they have not recovered. Work assessment allowance ensures further income (about 2/3 of previous income), and the period should be used for treatment and work rehabilitation. The work assessment allowance period can last for three years. The person can go on to receive disability pension if the person still has at least 50% reduced work ability after the work assessment period.

Employment is important for wellbeing. It can play a role in facilitating recovery from mental health problems (the enhancement hypothesis), and the mental health aspects are especially pronounced when compared with the effects of unemployment (Frijters et al., 2014; Gedikli et al., 2023; Modini et al., 2016; Plana-Ripoll et al., 2023; Rueda et al., 2012; Thomson et al., 2022; van der Noordt et al., 2014; Waddell and Burton, 2006). Benefits from working can include financial security, daily structure, social interaction, a sense of worth, autonomy, status, and personal development.

However, according to the occupational stress hypothesis, certain occupations may be more stressful than others. There has been considerable research on psychological effects of certain types of jobs such as shift work and healthcare workers, but few studies have investigated psychological profiles across different types of occupations, and the existing studies usually have minimal assessment of psychological complaints. Furthermore, these studies have not looked specifically at employment in treatment-seeking samples and how they might differ from the general population.

Job-related characteristics and certain occupations could be associated with psychological problems, but the association between employment, type of occupation, and mental health is complex and likely influenced by selection effects (e.g., occupational choice and selection out of certain jobs). Self-reported mental health problems have been associated with lower-paid occupations (e.g., elementary occupations, sales and customer service, and process-, plant-, and machine operatives), and the use of psychotropics with “public facing” occupations such as sales, administrative/secretarial, or caring roles (Ferry et al., 2023).

In the UK, higher rates of mental health problems have been observed among occupational groups such as managers and administrators, teachers, clerical and secretarial, sales, and personal and protective services (e.g., welfare and youth workers, and care assistants) (Stansfeld et al., 2011). It was suggested that these groups were characterized by emotional demands and lack of job security. The same research group found later that caring personal service occupations had the greatest risk of common mental health problems but work characteristics did not explain the increased risk (Stansfeld et al., 2013). They suggested that selection effects could partly explain higher rates in certain occupations.

Some studies have found no clear evidence of large variations in mental health across occupations (Marchand et al., 2003; Inoue et al., 2010), but one study found a higher prevalence of mental health problems among machine operators, laborers, and cleaners (Marchand, 2007). In Norway, more mental health problems were found among agricultural and fishery workers (Riise et al., 2003), and more depression among low-skill occupations (Sanne et al., 2003).

Severity of depression is associated with worse work performance. Cognitive and emotional symptoms of depression (impaired concentration, sadness, and self-criticism) seem related to presenteeism, while sickness absence is more related to somatic symptoms of depression such as sleep difficulties, changes in appetite, and psychomotor impairment (Johnston et al., 2019). Anxiety also affects work participation showing a deflated employment trajectory, and many people with anxiety disorders do not receive optimal mental health treatment (Waghorn and Lloyd, 2005). These findings correspond with a comprehensive review finding a positive association between unemployment and anxiety, depression, male sex, and younger age, while higher levels of education and social support could buffer the negative outcomes of job loss (Virgolino et al., 2022).

Findings from longitudinal studies suggest there is a reciprocal relation between unemployment and wellbeing over time (Gedikli et al., 2023), and there is evidence suggesting that increases in income probably has a causal effect on improved mental health (Thomson et al., 2022). Similarly, a 10-wave panel data study indicated a substantial causal effect of mental health on employment (Frijters et al., 2014). The effects of mental health on employment could be large, as a Danish registry study found that people with mental disorders lost an additional 10.5 years of working life compared with the general population (Plana-Ripoll et al., 2023).

The first aim of this study was therefore to describe employment status and type of occupation in a treatment-seeking sample from a community mental health clinic, and to compare this group with the general Norwegian population. It was expected that the sample would have higher rates of unemployment and disability benefits compared to the general population. The study’s second aim was to examine mental health profiles for employment status and for specific occupational groups in this clinical sample. We expected that different types of unemployment (being unemployed or receiving disability benefits or work assessment allowance) would be associated with worse mental health. No specific hypothesis was put forward on the association between specific occupations and mental health problems, as there have been mixed findings.

## Materials and methods

### Participants and procedure

The study used a cross-sectional design using pre-treatment data. Participants consisted of treatment-seeking adults at an outpatient community mental health service from one of the largest cities in Norway. All patients seeking treatment were invited to participate. Data collection, using an online portal, took place from September 2020 to October 2023, where 2,014 of 2,553 (78.9%) consented to participate. The project was approved by the Regional Committee for Medical and Health Research Ethics (reference number: REK 2019/31836), and the National Center for Research Data (reference number: NSD2020/605327).

There were no exclusion criteria for participation. Participants did not undergo diagnostic evaluations, but they self-reported rated their main problems as follows: Anxiety (20.2%), depression (20.2%), sleep (8.5%), trauma (5.6%), other (5.4%), physical health (2.6%), being a close relative to a patient (2.5%), victim of violence (2.1%), isolation (2.1%), work (2.0%), financial (1.6%), coping with everyday living (1.5%), drug abuse (1.5%), anger (1.2%), and problems with prescription drugs (0.7%). Some participants did not report any of these problems. A study of patients at the clinic showed that the they reported considerably lower health-related quality of life than the general population, and comparable to patient-groups from specialist mental health services (Lindberg et al., 2023). The most impacted quality of life domains were pain/discomfort, anxiety/depression, and problems with usual activities.

The study took place at an outpatient community mental health service clinic. The clinic had three major patient groups. The first consisted of people with mild to moderate mental health problems (67.6%). The second group was people with mental health problems in addition to more complex life-challenges (24.4%). The third group was people with addiction and mental health problems (8.0%). The first group referred themselves to the clinic and were given low intensity interventions such as group psychoeducation, group therapy, guided self-help, or one-to-one consultations (Lindberg et al., 2025). The second group was mainly referred by general practitioners or health workers in the specialist mental health services. In the third group, most patients contacted the service without a referral.

### Measures

Employment status was cross-referenced by the authors with type of income to ensure correct coding of employment. Participants were classified as either being: employed, student, “work-study” (both working and studying), unemployed, receiving work assessment allowance or other types of social benefits (unemployment benefit or financial assistance), receiving disability pension, or being a pensioner. Employed patients on sick leave were categorized as employed and not investigated as a separate category.

In Norway, people can receive sickness benefits for up to 52 weeks, and they can apply for work assessment allowance if they have not recovered. Work assessment allowance ensures further income (about 2/3 of previous income), and the period should be used for finding appropriate employment and treatment. The work assessment allowance period can last for three years but can be extended in some instances.

Classification of occupations was based on categories from Statistics Norway (ssb.no/en). The categories were: (a) military/undisclosed, (b) managers (e.g., politicians, office managers, company managers), (c) academic (including teaching, lawyers, university), (d) college degree jobs (engineers, health-related professions, technicians), (e) clerical (office workers, customer services), (f) sales and service, (g) farmers, fishery, forestry, (h) skilled trades (construction workers, electricians, plumbers), (i) machine and transport, and (j) “other” (including cleaners). Because of few participants reporting “military/undisclosed” (*n* = 4) or “farmers, fishery, forestry” (*n* = 5) as their occupation, these two groups were not included in further analyses.

The Patient Health Questionnaire-9 (PHQ-9; Kroenke et al., 2001) was used to measure symptom severity of depression. Scores on PHQ-9 range from 0 to 27 with higher scores indicating more severe depression and a suggested cutoff score of 10 points. Scores are typically interpreted as: 0–5 mild, 6–10 moderate, 11–15 moderate/severe, and 15+ severe. PHQ-9 has good psychometric properties (Brattmyr et al., 2022). Cronbach’s alpha was 0.85.

The Generalized Anxiety Disorder-7 (GAD-7; Spitzer et al., 2006) was used to measure symptom severity of anxiety. Scores on GAD-7 range from 0 to 21 with higher scores indicating more severe symptoms and a suggested cutoff score of 10 points. Scores are typically interpreted as: 0–4 minimal, 5–9 mild, 10–14 moderate, and 15+ severe. GAD-7 has good psychometric properties (Brattmyr et al., 2022). Cronbach’s alpha was 0.84.

The Work and Social Adjustment Scale (WSAS; Mundt et al., 2002) was used to measure everyday functioning. It consists of five items (work, home management, social leisure, private leisure, and close relationships). The items are rated using a 0 (*not at all impaired*) to 8 (*very severely impaired*) scale. Scores are typically interpreted as follows: 0–9 low impairment, 10–19 moderate impairment, 20+ severe impairment. Cronbach’s alpha was 0.82.

The visual analog scale of the EQ-5D-5L (EQ-VAS; Herdman et al., 2011) was used to assess participants’ health. The item asked participants to rate their overall health on a scale ranging from 0 (worst health) to 100 (best health).

Participants were asked to report how many units of alcohol they consumed per week (they were provided with examples of how units are measured). They were also asked (yes/no) to report use of psychotropic medications (anxiolytics and anti-depressants), and analgesics.

### Statistical analyses

Employment status and type of occupation were compared with the general Norwegian population in 2022 as described by Statistics Norway (ssb.no/en). To estimate the size of the difference in occupations and employment rates between the sample and the population (not including pensioners), ratios were calculated by dividing the rate of groups in the community sample by that of the general Norwegian population (e.g., employed; 44% in the sample/56.1% in general the population = 0.78).

Mental health profiles associated with employment status and specific occupations were examined using logistic regression analyses. The independent variables included use of psychotropics, alcohol, and scores on the PHQ-9, GAD-7, WSAS, and EQ-VAS. The results present both unadjusted models and models adjusted for age, sex, household income, and civil status (single vs. not single). The analyses compared scores for a specific employment status with the scores of the other employment statuses, e.g., employed participants vs. all other employment groups (including students, unemployed, work assessment allowance, disability pension, pensioners, and the “work-study” category). The same procedure was used for specific occupational groups, e.g., managers vs. all other groups (including academics, college degree jobs, clerical, sales/service, skilled trades, machine/transport, and an “other job” category). Finally, linear robust regression compared occupational groups and employment statuses on a composite score of the six independent variables representing mental health problems in general. The composite score was computed by averaging z-scores for all the six measures of mental health (PHQ-9, GAD-7, WSAS, EQ-VAS, use of psychotropics, and number of alcohol units). For psychotropic medication we used the number of drugs used including analgesics. There were few incidents of missing data (2.9% for data included in the employment status regressions, and 2.4% for the regressions comparing specific occupations with each other). Missing data were not imputed.

## Results

### Sample characteristics

Most participants reported to be living with a partner (42.9%) or alone (37.0%), while 16.6% lived with parents or others, and 3.5% had other arrangements. A total of 12.3% of the sample was born outside Norway (6.6% of the sample originated from a developing country). Previous treatments included: general mental health treatment (38.6%), mental health treatment at a community mental health clinic (17.3%), private practice (14.0%), occupational rehabilitation (8.4%), contact with child services (8.1%), addiction treatment (6.2%), and crisis shelters (3.9%). A total of 16.2% reported ongoing treatments at other health services.

Table 1 summarizes demographic and psychological characteristics of the major employment groups. A total of 43.4% were employed, while 20.2% reported to be both working and studying. The third largest group was participants receiving work assessment allowance or other social benefits (14.0%), while 9.5% had disability benefits. Students represented 9.0% of the sample, while 2.7% were unemployed, and 1.3% were pensioners. The sample reported worse general health on the EQ-VAS with a mean score of 53.0 (SD = 19.4) compared with the Norwegian general population’s score of 77.9 (SD = 18.3) (Garratt et al., 2022), a difference which equaled an effect size of *d* = 1.31.

**TABLE 1:** Demographic and psychological characteristics of employment status groups [M (SD)/%].

Variable	Total	Employed	Student	Work-study	Unemployed	Work assessment	Disability	Pensioner
*N* (%)	2,014 (100)	874 (43.4)	182 (9.0)	406 (20.2)	54 (2.7)	281 (14.0)	191 (9.5)	26 (1.3)
Age	36.42 (12.13)	37.92 (10.79)	25.92 (3.82)	33.30 (10.09)	29.09 (10.27)	34.52 (10.52)	46.12 (12.63)	71.88 (11.10)
Male	32.2	34.3	21.4	30.0	35.2	38.1	26.7	38.5
Single	58.2	47.0	72.0	61.5	79.6	69.5	69.8	44.0
Higher educ.	52.8	67.0	47.3	52.7	29.6	29.5	31.9	69.2
Prev. tx	60.3	47.9	70.9	54.4	55.6	85.1	84.3	57.7
Analgesics	13.6	9.4	8.0	11.4	20.0	17.8	35.7	8.0
Anxiolytics	10.1	6.2	5.7	5.4	10.0	18.3	27.1	32.0
Antidepr.	14.9	9.8	14.8	9.8	14.0	25.8	33.5	16.7
Alcohol	3.38 (10.62)	3.05 (5.71)	2.77 (4.70)	3.05 (4.95)	2.48 (4.05)	3.90 (10.64)	5.83 (28.19)	2.00 (2.24)
PHQ-9	13.65 (5.79)	12.86 (5.45)	13.49 (5.11)	13.05 (5.88)	14.21 (6.09)	15.63 (5.91)	15.75 (6.24)	13.16 (6.34)
GAD-7	11.40 (4.72)	10.79 (4.55)	11.47 (4.59)	11.33 (4.75)	12.31 (5.15)	12.36 (4.75)	12.61 (4.93)	11.16 (5.28)
WSAS	19.14 (8.70)	17.89 (8.28)	18.81 (7.87)	17.96 (8.59)	18.56 (8.78)	22.96 (8.47)	22.59 (9.15)	16.52 (10.48)
EQ-VAS	53.02 (19.41)	57.20 (18.25)	55.06 (17.67)	53.70 (18.92)	50.69 (18.49)	44.68 (19.26)	42.54 (20.29)	58.56 (19.10)

Higher education = completed more than high-school. Tx = treatment. Antidepr., antidepressants; PHQ-9, Patient Health Questionnaire-9; GAD-7, Generalized Anxiety Disorder-7; WSAS, Work and Social Adjustment Scale; EQ-VAS, visual analog scale from the EQ-5D-5L. Employed participants on sick leave were categorized as “Employed” (58.0% had no sick leave, 21.7% full-time sick leave, and 20.3% part-time sick leave). In the unemployed category, 12% also reported to be on sick leave (entitling them to sickness benefits rather than unemployment benefits for the duration of the sick leave).

Most employed participants reported to be working in sales and service (19.9%), academic jobs (18.4%), college degree jobs (18.2%), and clerical work (17.5%). Skilled trades represented 8.9%, 7.4% had “other” jobs, 7.2% were managers, and 2.4% worked with machines and transportation. Table 2 summarizes demographic and psychological characteristics of the different occupational groups.

**TABLE 2:** Demographic and psychological characteristics of specific occupational groups [M(SD)/%].

Variable	Manager	Academic	College degree job	Clerical	Sales/service	Skilled trades	Machine/ transport	Other
*N* (%)	63 (7.2)	161 (18.4)	159 (18.2)	153 (17.5)	174 (19.9)	78 (8.9)	21 (2.4)	65 (7.4)
Age	42.46 (10.44)	40.20 (10.32)	38.78 (9.63)	39.41 (11.71)	33.83 (9.85)	33.90 (10.60)	37.85 (9.22)	38.18 (11.55)
Male	38.1	25.5	19.5	29.4	38.5	79.5	76.2	21.5
Single	37.3	39.4	40.9	41.8	55.1	60.3	55.0	63.3
Higher educ.	71.4	98.8	98.7	79.7	37.4	16.7	9.5	35.4
Prev. tx	34.9	49.1	46.5	48.4	47.1	55.1	57.1	50.8
Analgesics	10.2	5.8	8.4	10.9	13.7	2.7	10.0	13.3
Anxiolytics	1.7	3.2	5.8	5.4	7.1	8.1	5.0	16.7
Antidepr.	5.1	9.0	8.4	10.9	8.4	12.3	10.0	18.3
Sick leave	55.9	44.5	44.8	30.6	40.8	39.2	45.0	48.3
Alcohol	3.69 (6.37)	3.10 (4.45)	2.02 (3.59)	2.91 (5.35)	3.17 (4.68)	3.63 (6.92)	7.05 (18.17)	3.08 (6.07)
PHQ-9	13.75 (4.80)	12.02 (4.99)	11.45 (5.36)	11.93 (5.52)	14.10 (5.42)	14.15 (5.63)	14.57 (5.08)	14.35 (5.71)
GAD-7	11.18 (4.42)	10.47 (4.55)	10.09 (4.38)	9.94 (4.70)	11.74 (4.51)	11.65 (4.15)	10.86 (5.40)	11.38 (4.43)
WSAS	19.60 (8.16)	18.09 (8.09)	16.30 (7.93)	16.89 (8.18)	18.61 (8.22)	19.70 (9.49)	20.90 (7.06)	17.11 (8.19)
EQ-VAS	55.17 (19.82)	59.42 (17.03)	58.73 (18.24)	58.69 (16.14)	55.29 (19.30)	55.97 (20.38)	52.35 (14.00)	54.56 (19.42)

Higher education = completed more than high-school. Tx = treatment. Antidepr., antidepressants; PHQ-9, Patient Health Questionnaire-9; GAD-7, Generalized Anxiety Disorder-7; WSAS, Work and Social Adjustment Scale; EQ-VAS, visual analog scale from the EQ-5D-5L.

### Comparisons with the general population

**FIGURE 1:**
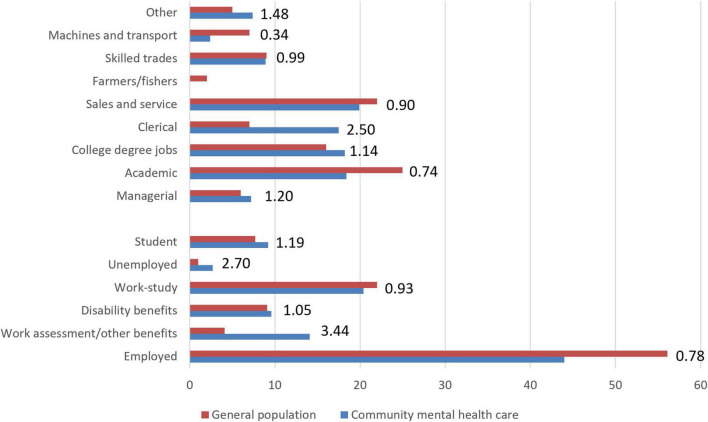
Comparisons between the sample and the Norwegian population on general employment categories and type of occupation. Numbers are presented as percentages. Numbers listed at the end of the bars represent the ratio between the sample and the general population (higher numbers [above 1.0] indicate larger rates in the community mental health sample). Data from the general population were collected from Statistics Norway and are for persons above 15 years of age in 2022. Pensioners were excluded from these analyses (they represented 21.5% of the general population and 1.3% in the community mental health sample).

Figure 1 compares employment categories and type of occupations for the community mental healthcare sample with the general Norwegian population. Groups overrepresented in the sample included unemployed participants (ratio = 3.44), people on work assessment allowance/other benefits (ratio = 2.70), and students (ratio = 1.19). The most underrepresented group in the sample was employed participants (ratio = 0.78). The sample was more comparable to the Norwegian population regarding disability benefits and other/unknown employment.

The type of occupation among participants was also compared with the general population. Clerical workers were overrepresented in the sample (ratio = 2.50), as were “other” occupations (ratio = 1.48). The sample had fewer participants working with machines and transport (ratio = 0.34) and in academic jobs (ratio = 0.74).

### Mental health profiles associated with employment status and type of occupation

Table 3 describes the mental health profiles for the different employment statuses. The employed group reported less use of psychotropics and better functioning and health. Students reported less use of psychotropics and slightly fewer symptoms of depression and anxiety. The “work-study” group reported less use of psychotropics, lower levels of depression, and higher functioning. The unemployment group reported more use of psychotropics. The disability group showed a clearly higher use of psychotropic medication (OR = 3.84), and reported more depression (OR = 1.05), anxiety (OR = 1.04), as well as lower functioning (OR = 1.04) and health (OR = 0.98). The work assessment allowance group reported a profile similar to the disability benefits group.

**TABLE 3:** Mental health profiles for employment statuses (Odds ratios and 95% CI).

Variable	Model	Employed	Student	Work-study	Unempl.	WAA	Disability
Psychotropics	^a^	0.45*** (0.36–0.55)	0.78 (0.54–1.12)	0.61*** (0.47–0.80)	1.24 (0.68–2.26)	2.22*** (1.70–2.90)	5.68*** (4.12–7.83)
	^b^	0.51*** (0.40–0.66)	0.65* (0.42–0.99)	0.60*** (0.45–0.80)	2.01* (1.05–3.86)	1.80*** (1.34–2.42)	3.84*** (2.68–5.51)
Alcohol	^a^	0.99 (0.98–1.00)	0.99 (0.96–1.02)	1.00 (0.98–1.01)	0.98 (0.92–1.04)	1.00 (0.99–1.01)	1.01 (1.00–1.03)
	^b^	1.00 (0.98–1.01)	0.99 (0.95–1.03)	1.00 (0.98–1.01)	0.95 (0.86–1.04)	1.00 (0.99–1.01)	1.01 (0.99–1.02)
PHQ-9	^a^	0.96*** (0.94–0.97)	1.00 (0.97–1.02)	0.98* (0.96–1.00)	1.02 (0.97–1.07)	1.07*** (1.05–1.10)	1.07*** (1.04–1.10)
	^b^	0.99 (0.97–1.01)	0.96** (0.92–0.99)	0.97* (0.95–0.99)	1.01 (0.96–1.07)	1.06*** (1.03–1.08)	1.05** (1.02–1.08)
GAD-7	^a^	0.95*** (0.93–0.97)	1.00 (0.97–1.04)	1.00 (0.97–1.02)	1.04 (0.98–1.11)	1.05*** (1.02–1.08)	1.06*** (1.03–1.10)
	^b^	0.98 (0.96–1.01)	0.96* (0.92–1.00)	0.99 (0.96–1.01)	1.02 (0.96–1.09)	1.04* (1.01–1.07)	1.04* (1.00–1.08)
WSAS	^a^	0.97*** (0.96–0.98)	1.00 (0.98–1.01)	0.98** (0.97–0.99)	0.99 (0.96–1.02)	1.06 (1.05–1.08)	1.05*** (1.03–1.07)
	^b^	0.98** (0.97–0.99)	0.98 (0.96–1.00)	0.98*** (0.96–0.99)	1.00 (0.97–1.04)	1.06*** (1.04–1.08)	1.04*** (1.02–1.06)
EQ-VAS	^a^	1.02*** (1.02–1.03)	1.01 (1.00–1.01)	1.00 (1.00–1.01)	0.99 (0.98–1.01)	0.97*** (0.97–0.98)	0.97*** (0.96–0.98)
	^b^	1.02*** (1.01–1.02)	1.01* (1.00–1.02)	1.00 (1.00–1.01)	0.99 (0.97–1.00)	0.95 (0.86–1.05)	0.98*** (0.97–0.99)

Psychotropics include analgesics.

^a^unadjusted model.

^b^Models adjusted for age, sex, household income, and civil status (single vs. not single). PHQ-9, Patient Health Questionnaire-9; GAD-7, Generalized Anxiety Disorder-7; WSAS, Work and Social Adjustment Scale; EQ-VAS, Visual analog scale from the EQ-5D-5L; WAA, work assessment allowance; Unempl., unemployed.

*p < 0.05.

**p < 0.01.

***p < 0.001.

Table 4 summarizes mental health profiles for specific occupational groups. The odds ratios were in general smaller than those found for employment status. In the adjusted models there were significant findings for the manager and college degree groups. Managers showed higher levels of depression (OR = 1.07) and worse work- and social functioning (OR = 1.04). The college degree job group showed less use of alcohol (OR = 0.95), less depression (OR = 0.94), less anxiety (OR = 0.97), and better work- and social functioning (OR = 0.97).

**TABLE 4:** Mental health profiles for specific occupational groups (Odds ratios and 95% CI).

Variable	Model	Manager	Academic	College degree	Clerical	Sales/service	Skilled trades	Machine/ transport	Other
Psychotropics	^a^	0.55 (0.25–1.25)	0.74 (0.46–1.19)	0.79 (0.50–1.27)	1.06 (0.68–1.66)	1.32 (0.88–2.00)	0.99 (0.54–1.83)	1.44 (0.51–4.01)	1.77 (0.98–3.19)
	^b^	0.57 (0.25–1.31)	0.83 (0.51–1.35)	0.79 (0.49–1.27)	1.23 (0.77–1.96)	1.22 (0.78–1.90)	1.14 (0.57–2.27)	1.12 (0.35–3.54)	1.25 (0.67–2.34)
Alcohol	^a^	1.02 (0.98–1.05)	1.00 (0.97–1.03)	0.93** (0.88–0.98)	1.00 (0.96–1.03)	1.00 (0.98–1.03)	1.02 (0.98–1.05)	1.05* (1.01–1.09)	1.00 (0.96–1.05)
	^b^	1.01 (0.97–1.05)	1.01 (0.98–1.04)	0.95* (0.89–1.00)	0.99 (0.96–1.03)	1.01 (0.98–1.04)	0.99 (0.95–1.04)	1.03 (0.99–1.07)	1.01 (0.96–1.05)
PHQ-9	^a^	1.03 (0.98–1.08)	0.97* (0.94–1.00)	0.94*** (0.91–0.97)	0.96* (0.93–0.99)	1.05*** (1.02–1.09)	1.05* (1.00–1.10)	1.06 (0.98–1.15)	1.06* (1.01–1.11)
	^b^	1.07** (1.02–1.13)	0.99 (0.95–1.02)	0.94** (0.91–0.98)	0.97 (0.94–1.01)	1.03 (0.99–1.06)	1.04 (0.99–1.09)	1.04 (0.95–1.13)	1.02 (0.97–1.08)
GAD-7	^a^	1.02 (0.96–1.08)	0.98 (0.94–1.02)	0.96* (0.92–1.00)	0.95* (0.91–0.99)	1.06** (1.02–1.10)	1.05 (0.99–1.10)	1.00 (0.91–1.10)	1.03 (0.98–1.09)
	^b^	1.06 (1.00–1.13)	1.01 (0.97–1.05)	0.96* (0.92–1.00)	0.97 (0.93–1.01)	1.04 (1.00–1.08)	1.04 (0.98–1.10)	0.97 (0.87–1.07)	0.99 (0.93–1.05)
WSAS	^a^	1.03 (1.00–1.06)	1.00 (0.98–1.03)	0.97** (0.95–0.99)	0.98 (0.96–1.00)	1.01 (0.99–1.03)	1.03 (1.00–1.06)	1.05 (0.99–1.11)	0.99 (0.96–1.02)
	^b^	1.04* (1.01–1.08)	1.02 (0.99–1.04)	0.97* (0.95–0.97)	1.00 (0.97–1.01)	1.00 (0.98–1.02)	1.03 (1.00–1.07)	1.03 (0.97–1.09)	0.97* (0.94–1.00)
EQ-VAS	^a^	0.99 (0.98–1.01)	1.01 (1.00–1.02)	1.01 (1.00–1.02)	1.01 (1.00–1.02)	0.99 (0.98–1.00)	1.00 (0.98–1.01)	0.99 (0.96–1.01)	0.99 (0.98–1.01)
	^b^	0.99 (0.97–1.00)	1.00 (0.99–1.01)	1.01 (1.00–1.02)	1.00 (0.99–1.01)	1.00 (0.99–1.01)	1.00 (0.98–1.01)	0.99 (0.97–1.02)	1.00 (0.99–1.02)

^a^unadjusted model.

^b^Models adjusted for age, sex, household income, and civil status (single vs. not single). Psychotropics include analgesics. PHQ-9, Patient Health Questionnaire-9; GAD-7, Generalized Anxiety Disorder-7; WSAS, Work and Social Adjustment Scale; EQ-VAS, visual analog scale from the EQ-5D-5L.

*p < 0.05.

**p < 0.01.

***p < 0.001.

In the unadjusted models there were indications that the machine/transport group used more alcohol. Unadjusted coefficients for depression showed lower scores for the academic and clerical groups, and higher scores for sales/service and skilled trades. For anxiety, there were also lower scores for the clerical group and higher for sales/service. There were no differences between occupational groups on use of psychotropics or on general health in both the adjusted and unadjusted models.

**FIGURE 2:**
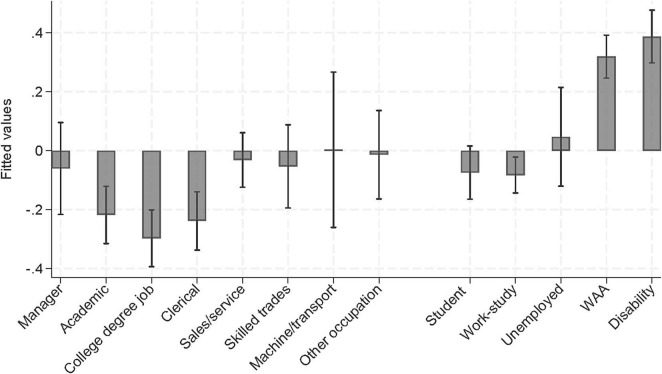
Predicted mental health composite scores for specific occupational groups and general employment statuses. WAA, work assessment allowance.

Linear robust regression of the composite score with specific occupational groups and employment statuses, showed that the work assessment allowance- and the disability benefits groups had significantly higher levels of mental health problems compared to all specific jobs. The comparisons are summarized in Figure 2. A *post hoc* analysis revealed that employed participants on sick leave reported poorer mental health compared to employed participants not on sick leave (*d* = 0.39), and those on part-time sick leave had better mental health than people on 100% sick leave (*d* = 0.55).

## Discussion

This study set out to explore associations between employment status, type of occupation, and mental health problems in a large sample seeking treatment at a community mental health clinic in Norway. Compared with the general population, the sample had higher rates of unemployed participants, more people on work assessment allowance/other benefits, and worse general health. The disability group and the work assessment allowance groups showed higher use of psychotropic medication, reported more anxiety and depression, as well as lower functioning and health. The analyses found worse depression and work- and social functioning among managerial jobs. People with college degree jobs (engineers, health-related professions, technicians) showed better mental health, but the effects were not large. In summary, the results suggested that type of occupation was less relevant to mental health, while different types of unemployment were associated with worse mental- and physical health.

As mental illness is one of the leading causes of sickness absence it is necessary with further research on how to help people with both aspects. Working is important for wellbeing (Rueda et al., 2012; van der Noordt et al., 2014; Waddell and Burton, 2006) and can play a role in facilitating recovery from mental health problems (the enhancement hypothesis), and the mental health aspects are especially pronounced when compared with the effects of unemployment (Modini et al., 2016). Rueda et al. (2012) found that employment can reduce symptoms of depression and anxiety, while van der Noordt et al. (2014) highlighted that being employed improves self-esteem and provides a sense of purpose and belonging. These findings align with our study, indicating a positive association between employment and better mental health, although it cannot establish causation between the two. Causation between mental health and employment is complicated and bi-directional effects are likely (Gedikli et al., 2023).

This study did not find any clear evidence that certain occupations was drastically more stressful than others (the occupational stress hypothesis). It is important to note that the farmer/fisher group was not represented, which is a group that has been associated with worse mental health in Norway (Riise et al., 2003). The low rate of farmers and fishers was likely due to the clinic being situated within a larger Norwegian city. Clerical workers were overrepresented in the sample but did not show a clear mental health profile compared with the other groups. The sample had fewer participants working with machines and transport and in academic jobs. The results should be interpreted with caution as the relation between mental health and occupation is complex and influenced by selection effects.

Occupations vary in terms of the job security, hazards, and psychosocial conditions (e.g., control, demands, support). These factors could also be linked with different effects of self-selection, socio-economic status, and education, which could also affect vulnerability to mental health problems. Successful return to work for people with emotional problems and long-term sickness absence (6+ months) is likely predicted by multiple factors related to work, family, social status, and medical conditions (Blank et al., 2008). To develop efficient treatments for enhancing work participation for this group has been difficult, but workplace- and clinical interventions are modestly associated with reducing number of sick-leave days (Nigatu et al., 2016).

It is likely that having a job is more strongly associated with mental health than what kind of job one has. The unemployment group reported more use of psychotropics, and the disability group and the work assessment allowance group showed more use of psychotropic medication, reported more anxiety and depression, as well as lower functioning and health. In contrast, the employed group reported less use of psychotropics, fewer symptoms, and better functioning and health. These findings correspond with previous research indicating that higher levels of anxiety and depression including use of psychotropics are associated with lower work performance (Johnston et al., 2019; Virgolino et al., 2022; Waghorn and Lloyd, 2005).

The OECD (2013) and the Norwegian Labor and Welfare Administration have pointed out that the Norwegian work assessment allowance system does not help people back to work, and the system was labeled as a “waiting list for disability pension” (Kann and Kristoffersen, 2014). It is also unclear whether clinically representative mental health treatment reduces sick leave (Lundqvist et al., 2023). Therefore, better interventions are needed. However, research has also suggested that individualized and structured rehabilitation programs can significantly improve work participation among individuals receiving work assessment allowance (Reme et al., 2015). Helping people at an earlier stage could be beneficial for preventing people from falling out of work as sick leave days tend to increase before starting treatment and decrease after treatment (Kausto et al., 2022; Lundqvist et al., 2025).

Another intervention that could help is the use of partial sick leave rather than full sick leave (Kausto et al., 2014). Predictors important for returning to work are important to consider when designing new interventions. In a sample of disability beneficiaries with mental illness, a positive, recent history of working was the strongest predictor of employment, but also fewer years on disability rolls and less physical health problems were important (Metcalfe et al., 2017). On the other hand, factors commonly considered barriers to employment, such as diagnosis, substance use, and hospitalization history, were not significant. However, work-related self-efficacy could be important for returning to work (Lagerveld et al., 2010), and a potential therapeutic indicator for clinicians (Gjengedal et al., 2021).

The work assessment allowance group represents an at-risk group for being excluded from the labor market and should be of interest for further research. To date, there has been little intersectoral collaboration between mental health services, housing- and vocational services in Norway. The OECD suggested that Norway should attempt implement early interventions, reduce waiting times, avoid fragmentation of services, and to implement work focus at mental health clinics (OECD, 2013). Another important suggestion was to add work status as a quality indicator of treatment supplementing measures of symptoms, quality of life, and functioning. The current study supported this as the results indicated significant associations between employment and mental health. Related research has also suggested that integrated multidomain approaches including healthcare provision, service coordination, and work accommodation can improve work functioning and reduce costs associated with work disability (Cullen et al., 2018). We also consider early intervention as crucial as a Norwegian study found that sickness absence increases dramatically while patients are on waiting list for mental health treatment (Lundqvist et al., 2025).

### Limitations

The study had different limitations that must be considered such as the cross-sectional design which cannot be used to draw causational inferences. The classification of occupations was also broadly categorical and did not reveal individual job stress. More accurate details of specific work demands, autonomy, and recognition at work could reveal other relevant findings. Independent of type of occupation some work-related factors could promote mental wellbeing, such as a sense of coherence (Schäfer et al., 2020). Sense of coherence entails a feeling of confidence that one’s internal and external environments are predictable, and that there is a high probability that things will work out. Family demands can also vary across occupational groups and is important to control for in future research (Ferry et al., 2023).

The study also relied on self-reported data. Inclusion of register-based data would improve the study as for instance self-reported mental health problems and prescription of psychotropics may differ significantly (Ferry et al., 2023). It should also be noted that comparisons with the Norwegian population included all people above 15 years, while the sample consisted only of people above 18 years. Another limitation was that the study did not have information on duration of sick leaves. Participants with both short-term and long-term sick leaves were therefore classified as “employed” in the analyses which could disguise potentially relevant effects of being on sick leave. Similarly, the dichotomous categories of employment status did not differentiate possible effects for people with part-time work.

## Conclusion

The results were more in line with the work enhancement hypothesis rather than the occupational stress hypothesis, as the results found better mental health among employed participants, but few differences between specific occupations. However, the study design did not allow for any causal inferences. However, related research suggested that work is usually good for both physical and mental health (Waddell and Burton, 2006). Occupational differences may exist among individuals with common mental health problems, but the relation between mental health and employment is likely complex. Poor mental health significantly impacts individuals and families by reducing adults’ ability to be actively employed. Therefore, investing in effective mental health interventions that also assist individuals returning to the workforce is essential. Future research should address treatments integrating mental health focus along with work focus (Nieuwenhuijsen et al., 2020; Slater et al., 2023), as improvement in work functioning does not always align with improvement in self-reported symptoms (Axén et al., 2020). However, highly effective treatment of depression could be associated with increased work participation (Solem et al., 2019).

## Data Availability

The raw data supporting the conclusions of this article will be made available by the authors, without undue reservation.
